# Improving Low-dose Cardiac CT Images based on 3D Sparse Representation

**DOI:** 10.1038/srep22804

**Published:** 2016-03-16

**Authors:** Luyao Shi, Yining Hu, Yang Chen, Xindao Yin, Huazhong Shu, Limin Luo, Jean-Louis Coatrieux

**Affiliations:** 1 Laboratory of Image Science and Technology, Southeast University, Nanjing, China; Key Laboratory of Computer Network and Information Integration (Southeast University), Ministry of Education, China; 2 Centre de Recherche en Information Biomedicale Sino-Francais (LIA CRIBs), Rennes, France; 3 Department of Radiology, Nanjing Hospital Affiliated to Nanjing Medical University, 210096, Nanjing, China; 4 INSERM, U1099, Rennes, F-35000, France; 5 Université de Rennes 1, LTSI, Rennes, F-35000, France

## Abstract

Cardiac computed tomography (CCT) is a reliable and accurate tool for diagnosis of coronary artery diseases and is also frequently used in surgery guidance. Low-dose scans should be considered in order to alleviate the harm to patients caused by X-ray radiation. However, low dose CT (LDCT) images tend to be degraded by quantum noise and streak artifacts. In order to improve the cardiac LDCT image quality, a 3D sparse representation-based processing (3D SR) is proposed by exploiting the sparsity and regularity of 3D anatomical features in CCT. The proposed method was evaluated by a clinical study of 14 patients. The performance of the proposed method was compared to the 2D spares representation-based processing (2D SR) and the state-of-the-art noise reduction algorithm BM4D. The visual assessment, quantitative assessment and qualitative assessment results show that the proposed approach can lead to effective noise/artifact suppression and detail preservation. Compared to the other two tested methods, 3D SR method can obtain results with image quality most close to the reference standard dose CT (SDCT) images.

Cardiac computed tomography (CCT) is widely recognized not only as a reliable and accurate tool for the diagnosis of coronary artery diseases but also as an efficient mean for planning and guiding surgical interventions^1^,^2^. Although the high image quality is generally acknowledged, radiation doses associated with this non-invasive modality have received serious concern^3^. Although the approximate effective radiation dose for a coronary angiography CT scan can be lowered to less 1 mSv based on recent reports^4^,^5^, many clinicians still show concerns over current levels of radiation exposures^6^. CT radiation dose is cumulative in lifetime, repeated CT scanning can significantly increase the risk of cancers^7^. Low dose CT (LDCT) should thus be considered in order to alleviate the harm caused by radiations for the patients with coronary artery disease (CAD). Many methods have been proposed so far to obtain LDCT images, among which the most practical and widely used method is to lower the X-ray tube current by reducing the mA (milliampere), but at a cost of a severe deterioration of the CT image quality due to increased quantum noise and artifacts^8^,^9^,^10^,^11^. In the past ten years, many other approaches have been developed to improve the quality of LDCT images. They can be generally divided into three categories: pre-processing approaches, reconstruction approaches and post-processing approaches.

The first category refers to the techniques that restore the raw projection data for the filtered-backprojection (FBP) reconstructions. In refs 12, 13, 14, different filtering methods have been proposed to suppress quantum noise in raw projection data. The second category includes iterative reconstruction approaches, which model the CT imaging as an ill-posed inverse problem and solve it by maximizing a regularized cost function using iterative optimizations^15^,^16^,^17^,^18^,^19^,^20^,^21^,^22^,^23^,^24^. Many prior options, for example the total-variation minimization^18^, the nonlocal prior reconstruction^19^,^20^, the edge-preserving prior^21^ and the prior image constrained compressed sensing (PICCS) algorithm^22^ have been proposed in the past decade. It has been well validated that iterative algorithms can significantly improve image quality while reducing CCT radiation dose^25^,^26^. However, researches on pre-processing and reconstruction approaches are often highly limited in practice for the lack of well-formatted projection data.

The third category includes post-processing methods, which can be directly applied on reconstructed images to suppress noise and artifacts. The main advantages of post-processing methods are that they avoid the high computation load inherent to iterative reconstructions and can be easily implemented in most current CT systems. The aim of post-processing approaches on LDCT images is to obtain images with visual appearances close to the corresponding SDCT (standard dose CT) images. However, since mottled noise and streak artifacts are distributed non-uniformly over the whole image after the back-projection process in FBP algorithm, they cannot be explicitly modeled by some specific distribution. Processing methods based on heuristic assumptions should be considered. In refs 27, 28, 29, several noise reduction filters were proposed to improve LDCT images. Chen *et al*. also applied a large-scale nonlocal means (LNLM) filter to improve abdomen LDCT images^30^. This LNLM method was further combined with a multi-scale directional diffusion strategy to reduce streak artifacts in thoracic CT images^31^.

A growing interest in sparse representations (SR) and dictionary learning was observed in the past decade^32^,^33^,^34^,^35^,^36^,^37^. Successful applications have been reported in medical imaging field^38^,^39^,^40^,^41^,^42^. Natural image patches often possess repetitive structure patterns, and these patterns can be sparsely presented by a linear combination of atoms from a redundant dictionary. On the other hand, the high frequency noise and artifacts are often in random and complicated structures, and cannot be sparsely represented by limited featured atoms in dictionary. In this paper, a 3D SR based processing is applied to improve low-dose cardiac CT images. The proposed approach is built based on the assumption that 3D anatomical features in CCT can be more sparsely represented by a well-trained global 3D feature dictionary than noise and artifacts. The proposed method obtains a significantly enhanced performance over the 2D SR methods in^41^,^42^ by extending the sparse representation from 2D features to 3D features. In the experiments presented below, clinical LDCT images of patients with CAD were used. By performing 3D SR algorithm on LDCT images, the radiation dose can be significantly reduced while maintaining good image quality. This study is an extension of our earlier work in ref 43. The structure of this paper is as follows: in Section 2, experimental materials and the proposed algorithm are described. Results are given in Section 3. Section 4 concludes this paper with a brief description of its contributions and states some open questions for future work.

## Materials and Methods

### Clinical Data and Processing Environment

The protocol of this study (data collection and post-processing) was approved by our institutional ethical review board of Southeast University, China. 14 patients were involved in the experiments. All these patients have given their written consent to the participation and received remuneration for it. A non-conflict of interest for this work was declared. The proposed method was carried out in accordance with the approved guidelines. The data were analyzed anonymously. All CT examinations were performed on a dual-source CT system (Somatom Definition Flash, Siemens Healthcare, Forchheim, Germany). This CT system is the second-generation, dual source CT equipped with two 128-slice acquisition detectors. Two low-dose techniques can be applied to reduce radiation of cardiac CT. They are prospectively electrocardiography (ECG)-gated step and shoot (SAS) mode and prospectively ECG-gated high-pitch mode (also known as Flash Cardio mode). In^44^, experimental results show that both the SAS and high-pitch mode for low-dose CT coronary angiography provide a high accuracy for the assessment of significant coronary stenosis, while the high-pitch mode further significantly lowers the radiation dose. In this paper, both high-pitch mode and SAS mode are considered. In high-pitch mode, data acquisition is prospectively triggered with the ECG signal of the patient. The data are acquired in a spiral mode while the table translates with a very high pitch of 3.4 mm, which equals to a table feed of 46 cm/s. Under such a high-pitch, the entire heart can be scanned within a single cardiac cycle. However, the limitation of high-pitch mode is that a high diagnostic accuracy can be obtained only in patients with heart rates ≤60 bpm^45^. In SAS mode, data acquisition is also prospectively triggered with the ECG signal. In the SAS mode, x-ray tube is turned on only at a predefined time point in the cardiac cycle. The x-ray exposure time is shorter, and thus lower radiation can be obtained.

The patient cohort includes 6 women and 8 men with an average age of 57 years (age range: 34–85 years). All the patients suffer from cardiovascular disease. Seven of them took high-pitch mode scans and the other seven took SAS mode scans. Nitroglycerin spray was administered sublingually before scanning. The circulation time was measured before data acquisition. For high-pitch mode, test bolus method^46^ was used, and 15 mL of contrast media was injected at a rate of 5 mL/s. The circulation time was measured with serial scanning of the same slice in the ascending aorta to obtain the peak enhancement time through time density curve analysis. Then 60 mL of contrast agent (Ultravist, Iopromide, 370 mg/ml) was injected at a flow rate of 5 mL/s followed by 50 mL of saline solution. Data acquisition was initiated with a delay of 5 s after reaching peak enhancement time. For SAS mode, bolus tracking method^46^ was used to measure circulation time. 60 mL of contrast agent (Ultravist, Iopromide, 370 mg/ml) was injected at a flow rate of 5 mL/s followed by 50 mL of saline solution. Monitoring scans at the level of the ascending aorta were performed 8 seconds after the start of contrast agent injection. When the CT HU of the ROI reaches 100 HU, data acquisition is activated after a delay of 5 s.

Under high-pitch scan mode, SDCT and LDCT images were collected using a routine tube current 300 mA and reduced tube current 76 mA, respectively. For SAS mode, they were 240 mA and 80 mA, respectively. For each patient, the SDCT images were scanned first and the LDCT images were scanned six months later with same contrast agent (as in the SDCT scans). 270 slices were collected for each patient scan. Other scan parameters include: kVp, 120; slice thickness, 0.75 mm. We recorded the accumulated doses from the workstation for each scan with 270 slices. The recorded doses under high-pitch mode were 1.7 mSv for the routine 300 mA protocol, and 0.425 mSv for the low dose 76 mA protocol. For SAS mode, the doses were 7.65 mSv for the routine 240 mAs protocol, and 2.55 mSv for the low dose 80 mAs protocol. The CT images were reconstructed with an averaged field of view (FOV) of 185 mm. The FOV may differ slightly, depending on different patient cases. The CT image size is 512 × 512, which means that the averaged resolution is 2.768 pixels/mm. All the CT images were exported as DICOM files and then processed offline under a notebook computer (Intel Core™ i7-3630 QM CPU @ 2.40 GHZ and 8192 Mb RAM) with MATLAB as the developing language (MATLAB R2012a software; MathWorks).

### Method and Parameter Setting

Assuming the patches in the target LDCT image are sparsely representable, the patch based SR approach can be carried out by coding each patch as a linear combination of only a few patches in a dictionary^33^. This method finds a best global over-complete dictionary and then represents each image patch as a linear combination of a few dictionary vectors (atoms). The coefficients of the linear combination can be estimated through the sparse coding. Based on Elad’s work^35^, the SR approach can be applied to improve LDCT images by solving Eq. (1):


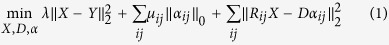


where *X* and *Y* denote the processed (and unknown) image and the original LDCT image, respectively. The subscript *ij* indexes each image pixel (*i*, *j*). *R*_*ij*_ represents the operator that extracts the 2D patch *x*_*ij*_ of size 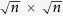 (the image patch with its top-left corner located at position (*i*, *j*) is extracted from image *X*). The patch-based dictionary *D* is an *n* × *K* matrix, where *K* is the number of atoms in the dictionary and each n-vector column corresponds to one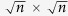 patch. *α* denotes the coefficient set for all the sparse representations of patches, and each patch can be approximated by a linear combination *Dα*_*ij*_. The 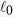 norm 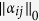 counts the nonzero entries of vector *α*_*ij*_. *μ*_*ij*_ is the weight of 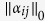. Solving Eq. (1) leads to the following two steps Eq. (2) and Eq. (3):


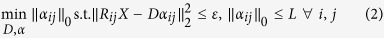



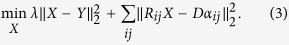


Here, Eq. (2) aims to train the coefficients *α* and dictionary *D* from a set of image patches and can be efficiently solved by the K-means Singular Value Decomposition (K-SVD) with the replacement of *X* by the observed image *Y*. Starting from an initial dictionary (e.g. the DCT dictionary, which is obtained by sampling the cosine wave functions in different frequencies), this K-SVD operation estimates *α* and *D* by alternatively applying two steps: (i) the Sparse Coding Step using the orthogonal matching pursuit (OMP) algorithm^47^,^48^,^49^ and (ii) the Dictionary Update Step based on SVD decomposition. The OMP algorithm is a matching pursuit algorithm used to solve the NP-hard sparse approximation problem. Note that the mean values of all the input patches are subtracted before representation, and added back to each patch in Eq. (3) to solve the represented image. The columns of the target dictionary *D* are constrained to be of unit norm to avoid scaling ambiguity in calculation. The *ε* in Eq. (2) denotes the tolerance parameter used in calculating *α* by the OMP method and is routinely modulated with respect to noise/artifact level in the LDCT images. Parameter *L* restricts the maximum atom number to a certain number in each representation, even if the tolerance constraint is not met. Then, with fixed dictionary *D* and *α*, we can obtain the output image 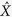 by solving Eq. (3) through zeroing the first order derivative with respect to image *X*:


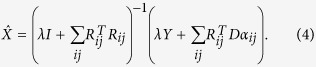


For the cardiac CT in this study, the data to be processed is in 3D volume instead of 2D slices. The 2D patch *x* in section 2.1 is replaced by a 3D block *x*^*V*^ of size 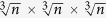. The 3D dictionary *D*^*V*^ is still an *n* × *K* matrix, but here each n-vector column corresponds to one 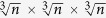 atom. The dictionary can be trained from LDCT data itself, but pre-training the dictionary from other available SDCT data before processing can significantly save computational costs without compromising the processing performance^41^. In the 3D case, with a pre-calculated dictionary 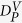 (obtained via solving Eq. (2) using other available SDCT volumes) chosen as the global dictionary, the proposed 3D SR processing algorithm is composed of the two steps:


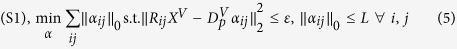



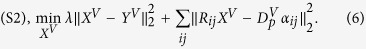


The sparse coefficient *α* can be solved via Eq. (5) using the OMP algorithm. The output image 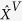 in Eq. (6) can be calculated via the above Eq. (4).

**Figure 1:**
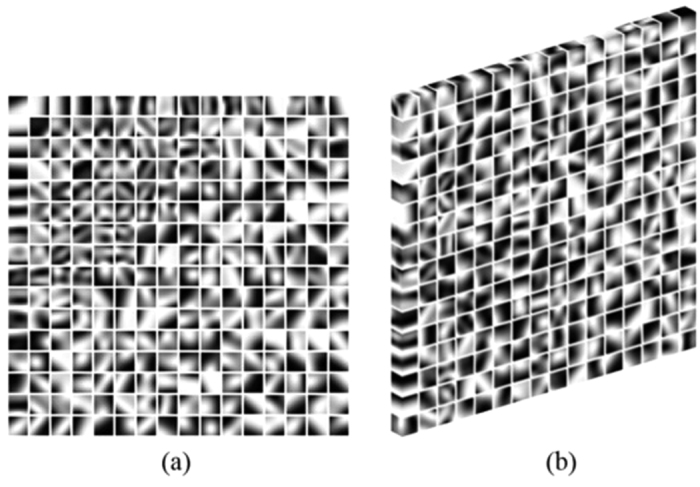
Illustration of 2D and 3D dictionaries. (**a**) a 2D dictionary (size 64 × 256) trained from 50 slices of 2D CCT images; (**b**) a partial 3D dictionary (1/4 atoms from a whole 3D dictionary of size 512 × 1000) trained from 5 patients’ CCT volume data.

The 3D approach is expected to perform better than the 2D approach based on the assumption that compared to noise and streak artifacts, 3D anatomical features in CCT can be more sparsely represented by 3D atoms from a 3D dictionary. Figure 1(a) shows a 2D dictionary (size 64 × 256) trained from 50 slices of 2D CCT images, and Fig. 1(b) shows a partial 3D dictionary (1/4 atoms from a whole 3D dictionary of size 512 × 1000) trained from 5 patients’ CCT volume data. Both the 2D dictionary and the 3D dictionary consisted of low frequency cartoon-like atoms (feature atoms), so that high frequency noise and artifacts cannot be sparsely represented by these atoms. Nevertheless, for some streak artifacts with strong intensity, the shapes of artifacts resemble anatomical structures and such artifacts could also be sparsely represented by 2D feature atoms. However, noise and artifacts are less correlated between slices, so the possibility that the noise and artifacts in a 3D block are sparsely represented by low frequency 3D feature atoms is very low. The 3D approach can thus remove noise and streak artifacts more effectively than the 2D approach.

We compared the proposed method with the 2D SR method and the state-of-the-art noise reduction algorithm BM4D, which suppresses noise by a grouped averaging operation weighted by patch similarities^50^. The parameters involved in the three methods were specified under the guidance of one radiological doctor (with 15 years of experience) to provide the best visual results. In the BM4D method, the ‘modified parameter profile’ was utilized (as explained in the paper^50^), and the noise variance parameter *σ* was set to 3%. The parameter settings for the 2D and 3D SR methods are listed in Table 1, and more details on these parameters can be found in Section 2 above.

### Quantitative methods

The LDCT and the corresponding SDCT images have no exact spatial correspondence to each other because of the inevitable displacements caused by patient heart beats, breath and movements in different data acquisitions. This makes it impossible to evaluate image quality using some Euclidean distance metrics (e.g. the mean squared error (MSE)) directly. We chose to compare the CT values, image noise, signal-to-noise ratio (SNR), and contrast to noise ratio (CNR) in the original and processed LDCT images with respect to those of the SDCT images.

The CT number is defined as the mean value of the selected regions of interest (ROI) and was expressed in Hounsfield Units, whereas image noise was measured by standard deviation (SD). Both CT number and image noise were measured from four circular ROIs placed at the ascending aorta, left main coronary artery, proximal right coronary artery and perivascular fatty tissue with sizes of 200, 8, 3 and 50 mm^2^, respectively^51^. The ROIs were selected under the guidance of the same doctor who guided the parameter specification. The measures were performed in an identical manner for all the four groups of images (LDCT, SDCT, 2D and 3D SR processed images).The SNR was calculated by dividing the averaged CT Hounsfield unit by the noise metrics SD while CNR was calculated by dividing the contrast enhancement (averaged CT HU at aorta or coronary artery minus CT HU at the fat) by the noise metric SD^51^,^52^, as given in Eq. (7) and Eq. (8):


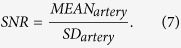



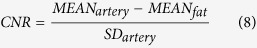


where *MEAN*_*artery*_ and *SD*_*artery*_ denote the mean values and standard deviations of the intensities within the ROIs placed in the aorta and coronary arteries. *MEAN*_*fat*_ denotes the mean values of the intensities within the ROIs placed in the fat tissue.

## Results

### Visual Assessment

**Figure 2:**
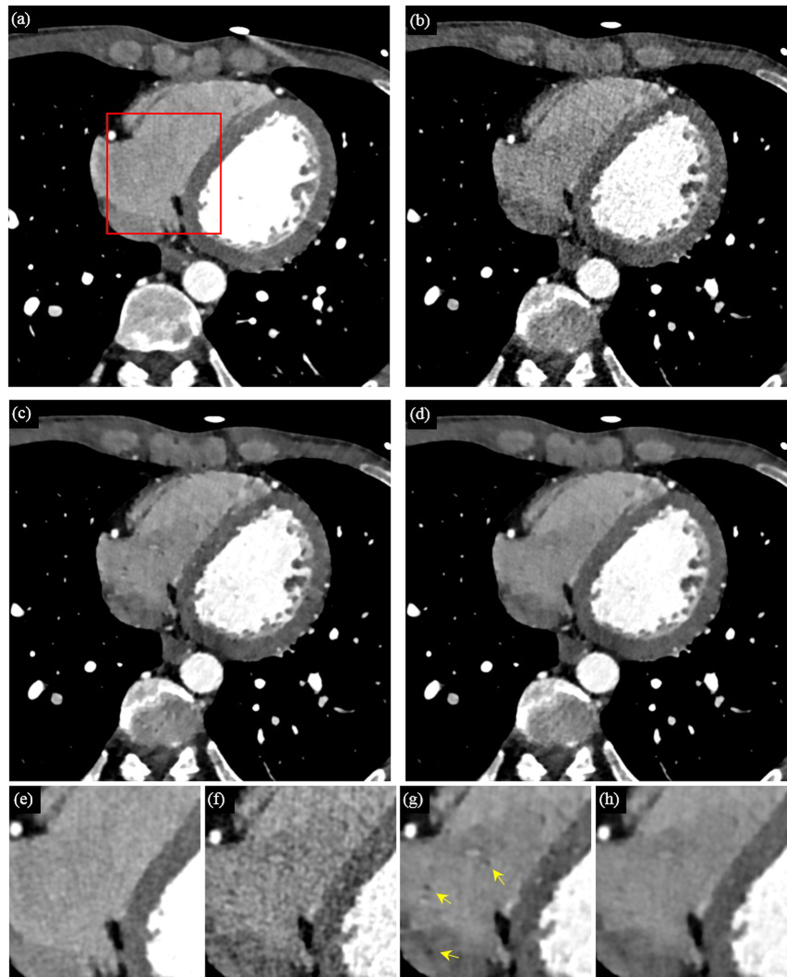
Processing results of one CCT image under high-pitch mode. (**a**) the reference SDCT image; (**b**) the original LDCT image; (**c**) 2D SR processed LDCT image; (**d**) 3D SR processed LDCT image; (**e**–**h**) show the zoomed ROI specified in (**a**).

**Figure 3:**
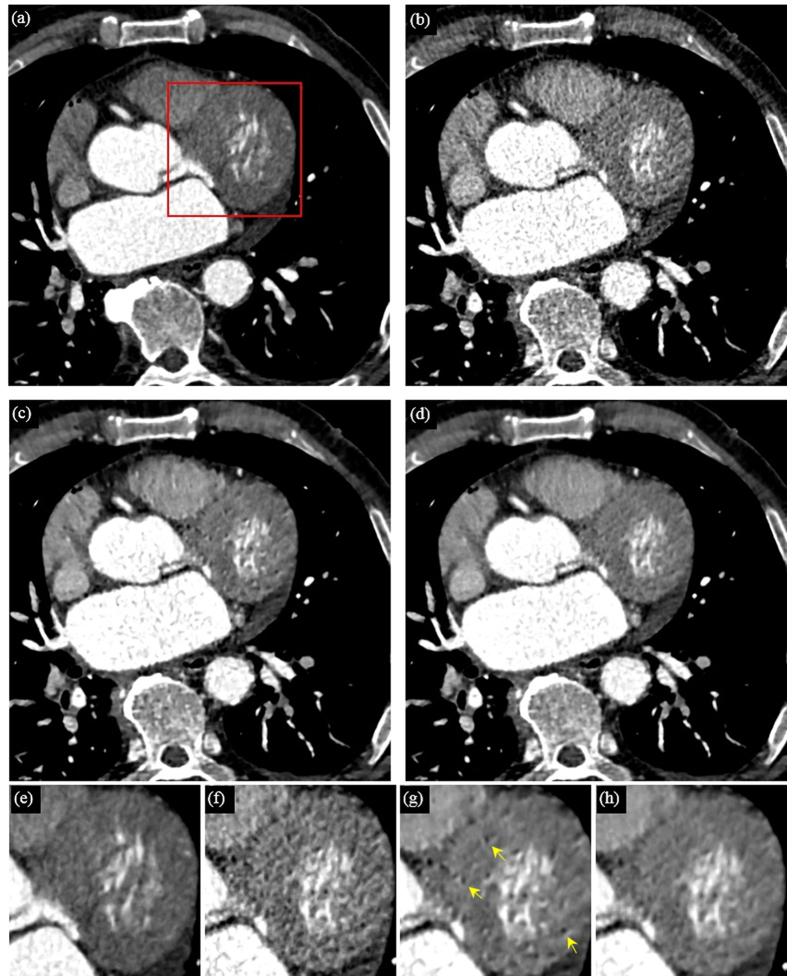
Processing results of one CCT image under step-and-shoot mode. (**a**) the reference SDCT image; (**b**) the original LDCT image; (**c**) 2D SR processed LDCT image; (**d**) 3D SR processed LDCT image; (**e**–**h**) show the zoomed ROI specified in (**a**).

In this part, all the CT images in axial view are shown in the CT angiography window (Level 150 HU, Window 450 HU). Figures 2 and 3 show the processing results of a CCT image under high-pitch mode and SAS mode, respectively. Figures 2(a) and 3(a) are the reference SDCT images. Figures 2(b) and 3(b) are the original LDCT images. The reference SDCT images were collected from a consecutive scan and selected from the slices which best match with the LDCT images. The results of the 2D SR method and the proposed 3D SR method are given in (c) and (d) in Figs 2 and 3, respectively. In Figs 2 and 3(e–h) illustrate the zoomed regions of interest (ROI) specified by the red squares in (a). By comparing (a) and (b) in Figs 2 and 3, we can see that, under LDCT scanning condition, mottle noise and streak artifacts severely degrade the reconstructed images and lower tissue discrimination. We can find in Figs 2(c) and 3(c) that the 2D SR method works well in mottle noise removal but remains yet ineffective in suppressing streak artifacts (see the residual artifacts indicated by yellow arrows). It can be observed in (d) in Figs 2 and 3 that our 3D SR approach performs much better in noise and artifact suppression, and can produce images with visual illustration similar to the reference SDCT images in Figs 2(a) and 3(a).

**Figure 4:**
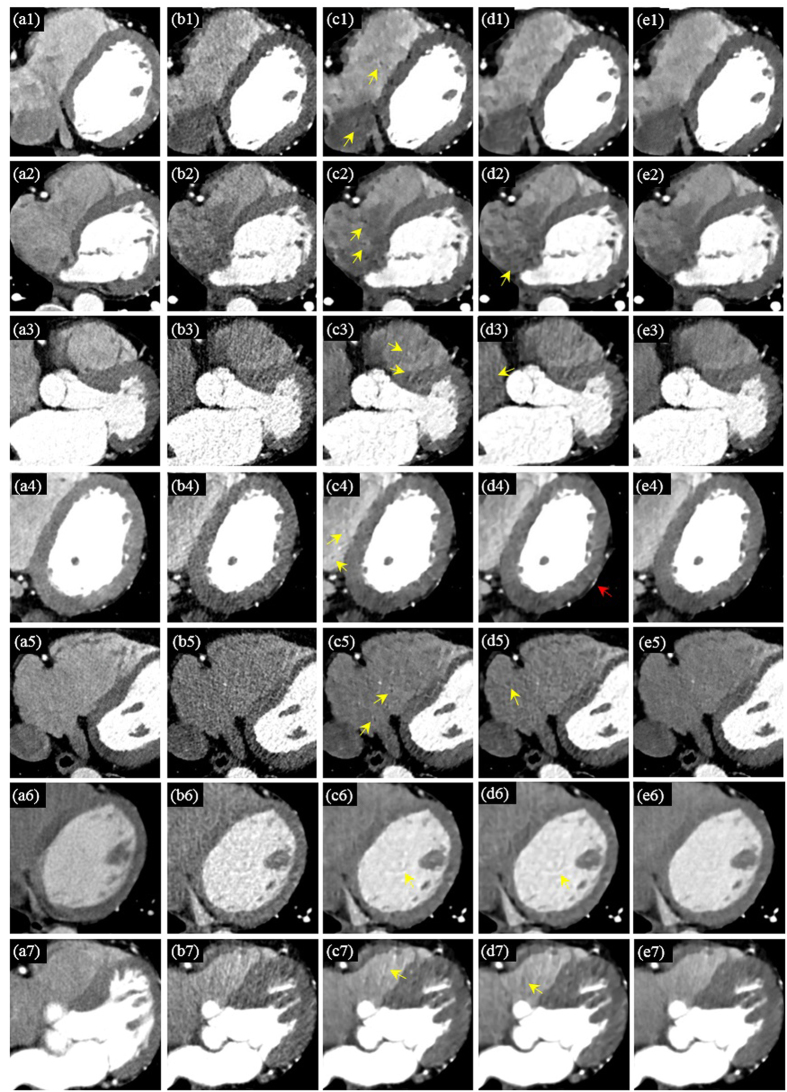
Processing results for CCT images under high-pitch mode of another seven adult patients. The first, second, third, fourth and fifth columns correspond to the reference SDCT images (a1–a7), the original LDCT images (b1–b7), the 2D SR processed LDCT images (c1–c7), the BM4D processed LDCT images (d1–d7) and the 3D SR processed LDCT images (e1–e7). The residual artifacts in the 2D SR and BM4D processed results are indicated by yellow arrows. The red arrow shows the blurred vessels in one BM4D processed image.

**Figure 5:**
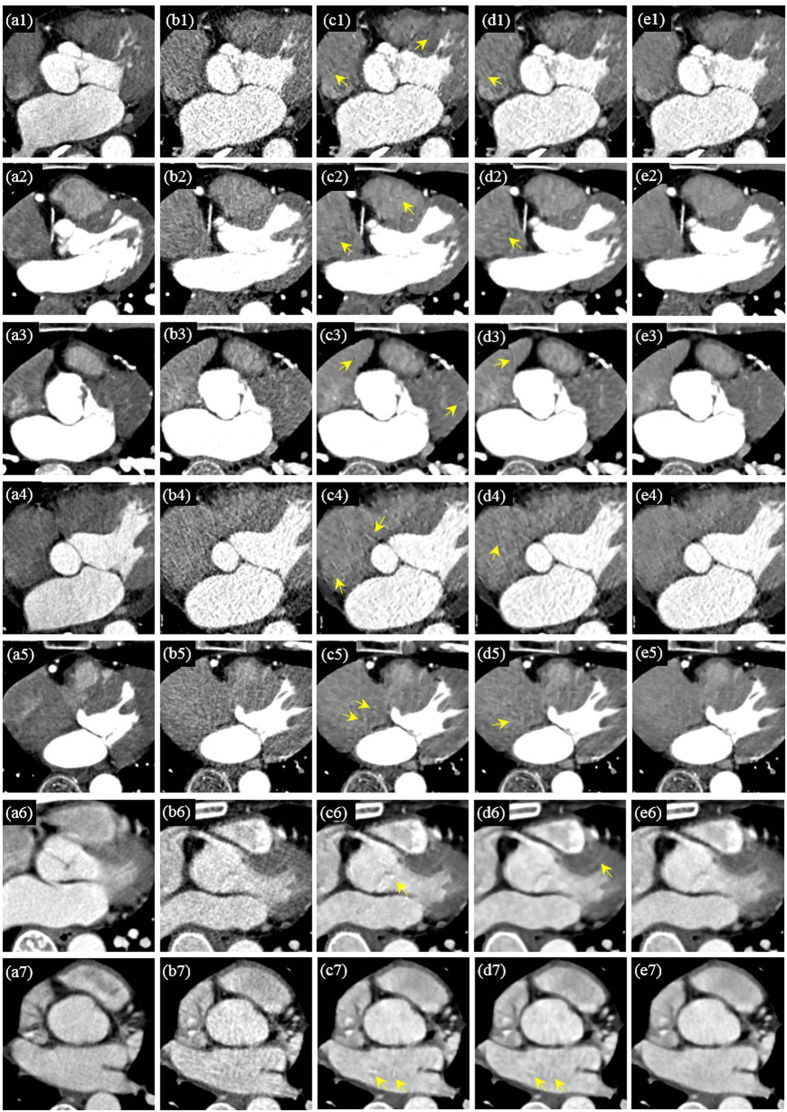
Processing results for CCT images under step-and-shoot mode of another seven adult patients. The first, second, third, and fourth columns correspond to the reference SDCT images (a1–a7), the original SDCT images (b1–b7), the 2D SR processed LDCT images (c1–c7), the BM4D processed LDCT images (d1–d7) and the 3D SR processed LDCT images (e1–e7). The residual artifacts in the 2D SR and BM4D processed results are indicated by yellow arrows.

Figure 4 provides the processing results for CCT images under high-pitch mode of seven adult patients and Fig. 5 the results for CCT images under SAS mode of another seven adult patients. All the images are given as zoomed image sections. The first, second, third, fourth and fifth columns correspond to the reference SDCT images (a1–a7), the original LDCT images (b1–b7), the 2D SR processed LDCT images (c1–c7), the BM4D processed LDCT images (d1–d7) and the 3D SR processed LDCT images (e1–e7). In Figs 4 and 5, with the SDCT images as reference, we observe that the mottle noise and streak artifacts severely degrade the LDCT images, and the 2D SR algorithm leads to smoother textures but with obvious noise/artifact residuals (as pointed by yellow arrows) in the processed LDCT images. The BM4D method provides strong noise suppression, but some large-scaled artifacts can still be observed in the processed images (see the yellow arrows in the fourth column in Figs 4 and 5). Additionally, some details were blurred in the BM4D processed results (see the blurred vessels pointed by red arrow in Fig. 4 (d4)). As to the BM4D method, a more aggressive parameter setting may further suppress noise and artifacts, but will also obscure more anatomical details. In the fifth column in Figs 4 and 5, we can see that the proposed 3D SR approach leads to effective noise/artifact suppression and detail preservation which also presents cardiac tissues in similar textures as the reference SDCT images.

**Figure 6:**
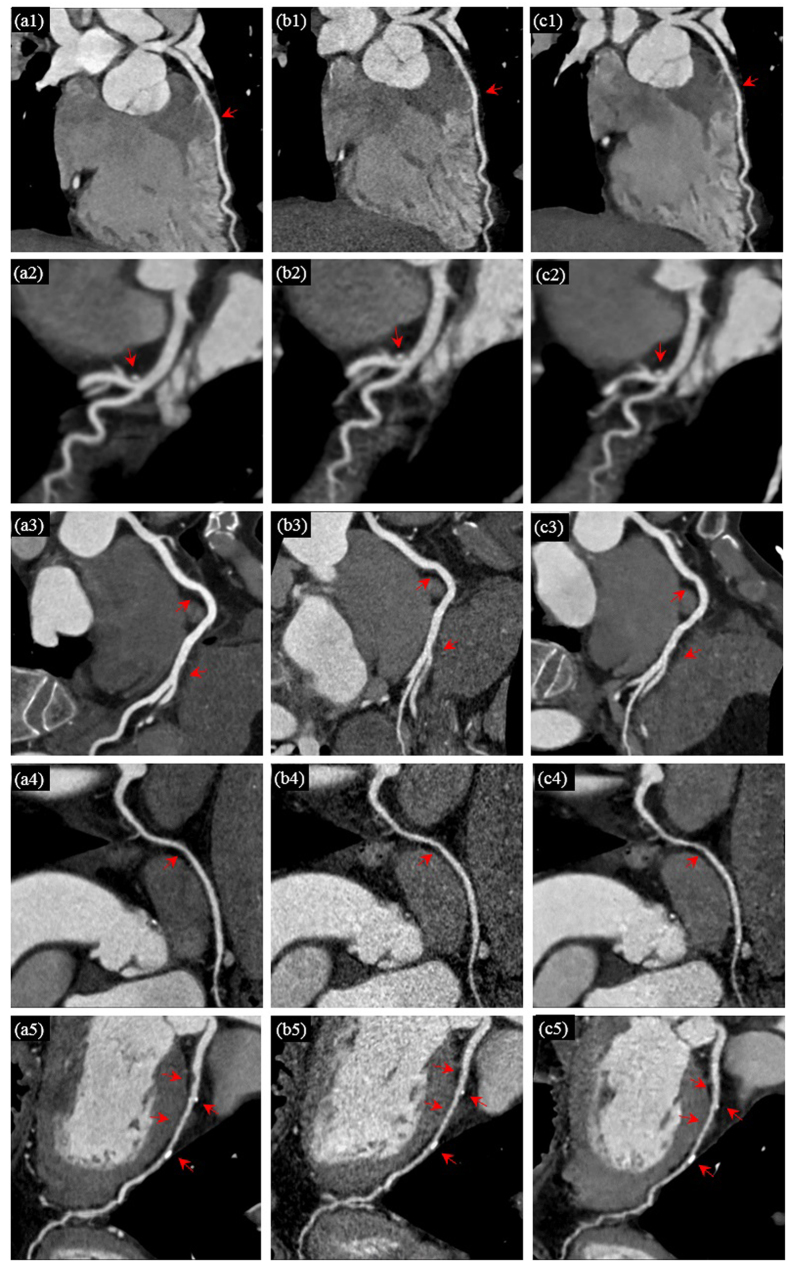
Curved planar reformatted CT images of five patients with vascular abnormalities. The first, second and third columns correspond to the CPR images derived from reference SDCT data, the LDCT data and the 3D SR processed LDCT data. The first row shows left anterior descending artery (LAD) with coronary myocardial bridge and mild stenosis (arrows); the second row shows anterior descending with hybrid plague and moderate stenosis (arrows); the third and fourth row show right coronary artery (RCA) with non-calcified coronary plaque and mild stenosis (arrows); the fifth row shows left anterior descending artery (LAD) with multiple calcified and non-calcified coronary plague, mild to moderate stenosis and coronary myocardial bridge (arrows).

In clinical setting, curved planar reformation (CPR)^53^ and three-dimensional volume rendering (VR)^54^ are frequently used by physicians. Curved planar reformations depict the cross-sectional profile of a vessel along its length while preserving the relative x-ray attenuation information, which allows reviewing the whole cross section within a single 2D image. Vascular abnormalities (i.e., stenosis, occlusions, aneurysms and vessel wall calcifications) can be so better investigated by physicians. Figure 6 provides the CPR images of five patients with vascular abnormalities for visual comparison. In Fig. 6, the first, second and third columns correspond to the CPR images derived from the reference SDCT data (a1–a5), the LDCT data (b1–b5) and the 3D SR processed LDCT data (c1–c5). More specifically in Fig. 6, the first row depicts the images of the left anterior descending artery (LAD) with coronary myocardial bridge and mild stenosis (pointed by red arrows); the second row illustrates the images of the anterior descending artery with hybrid plaque and moderate stenosis (pointed by red arrows); the third and fourth rows show the images of the right coronary artery (RCA) with non-calcified coronary plaque and mild stenosis (pointed by red arrows); the fifth row displays images of the left anterior descending artery (LAD) with multiple calcified and non-calcified plaques, mild stenosis and a coronary myocardial bridge (pointed by red arrows). It should be noted that CPR images of all the high-dose, low-dose and processed low-dose CT images were manually made by the radiologists. The radiologists often cannot produce the CPR images with identical locations and scales for the same set of original and processed images. So the CPR images with the best matches among different datasets are selected for illustration in Fig. 6. In the CPR images built from the 3D SR processed LDCT images, both noise and artifacts are found significantly suppressed, leading to a better visibility of vascular abnormalities.

**Figure 7:**
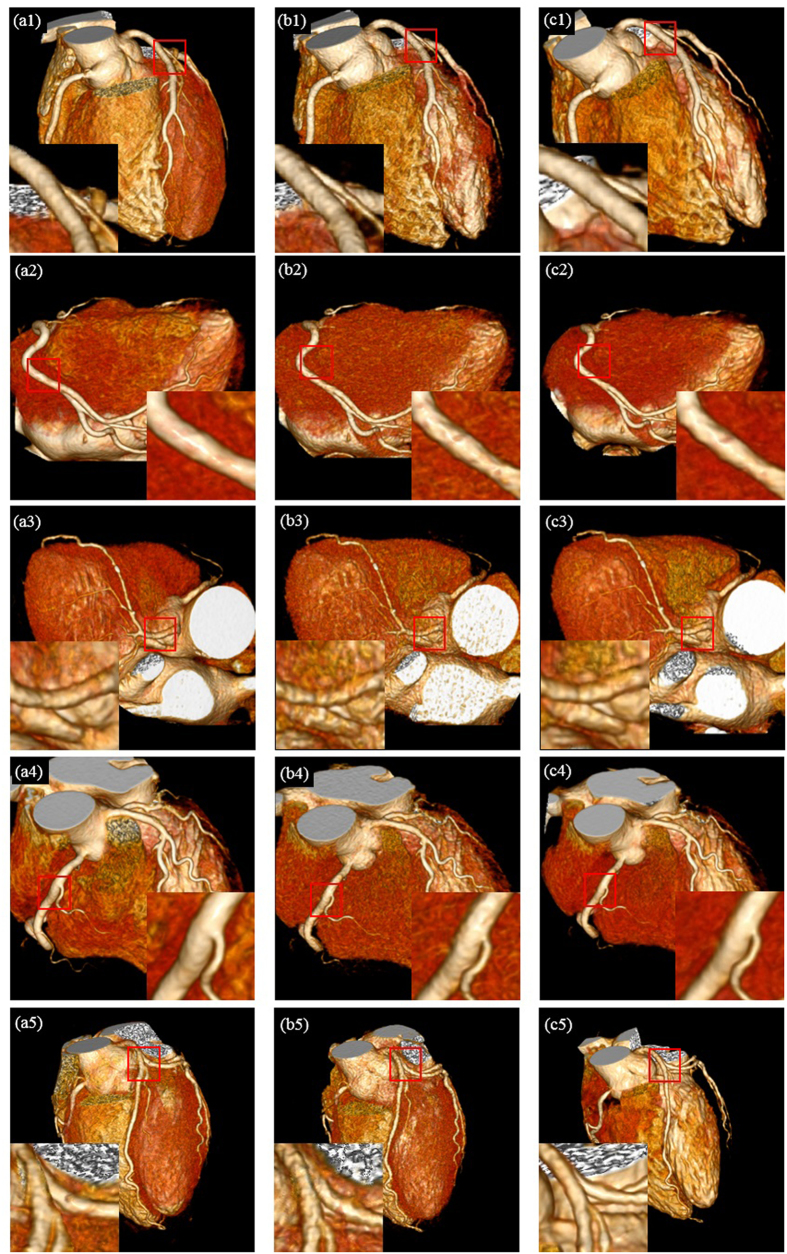
Volume rendering images of five patients. From above to bottom, the 1–3 columns correspond to the VR images of the reference SDCT, the original LDCT, and the 3D SR processed LDCT images, respectively. Each row corresponds to one patient case. Zoomed vessel surfaces are also provided in the lower parts in the images.

Volume rendering (VR) techniques can be used to transform serially acquired axial CT image data into 3D images. They provide additional insights to the vascular anatomy and a more comprehensive understanding of pathologic states. 3D volume rendering enables radiologists to efficiently and comprehensively review large data set and thus improve patient care. Such VR images are presented in Fig. 7. The three columns n Fig. 7 correspond to the VR images of the reference SDCT ((a1)–(a5)), the original LDCT ((b1)–(b5)), and the 3D SR processed LDCT images ((c1)–(c5)), respectively. Each row corresponds to one patient case. Details of the vessel surfaces are illustrated in zoomed images at the lower parts of the original images. Coronary arteries with rough and discontinuous surfaces can be observed in the VR results of the original LDCT images, as a consequence of noise and artifacts. In contrast, the 3D SR processed VR results show more smooth vessel surfaces with closer visual appearances to those of the original SDCT images.

### Quantitative Assessment

**Figure 8:**
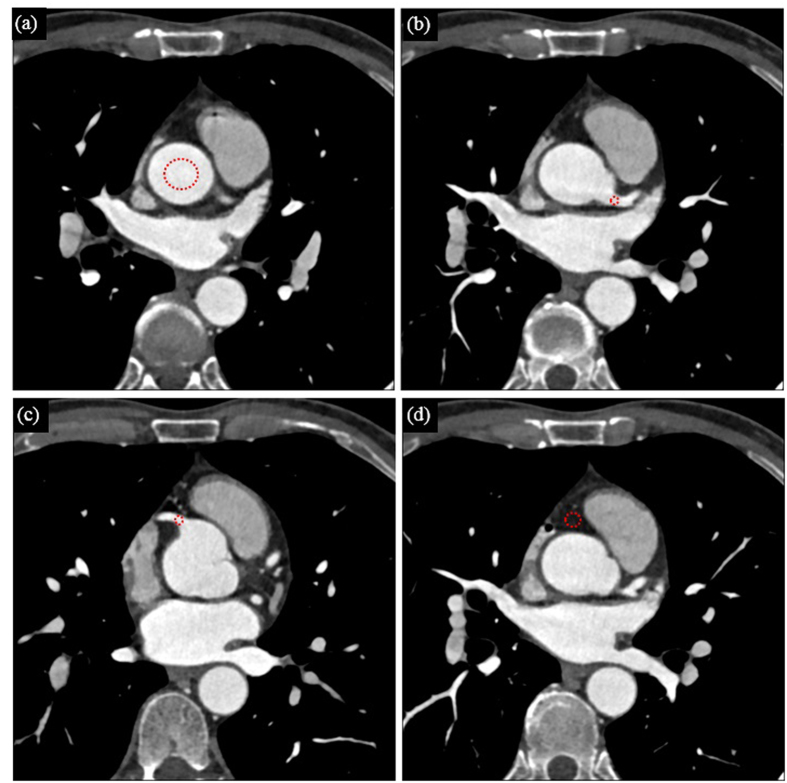
Four different regions of interest (ROI) with a circular ROI of 200 mm^2^, 8 mm^2^, 3 mm^2^ and 50 mm^2^ placed at the ascending aorta (**a**), the left main coronary artery (**b**), the proximal right coronary artery (**c**) and the perivascular fatty tissue (**d**), respectively.

Table 2 lists the mean CT number, the image noise, the calculated SNR and the CNR of the considered ROIs (described in the above section of Quantitative methods) as shown in Fig. 8. BOLD fonts are used to represent the best performance among the three processing methods (2D SR, BM4D and 3D SR) for each index. Seven images scanned under high-pitch mode and seven scanned under SAS mode were involved in this evaluation. One image was selected from the acquired images from each scan. It can be noticed that the mean CT numbers of the original and processed LDCT images are slightly different from those of the SDCT images, due to the fact that the contrast evolutions are not identical in the data acquisitions of the LDCT images and SDCT images. In Table 2, the mean CT numbers of the processed CT images are thus compared to the corresponding LDCT images (the BOLD fonts in the mean CT number category means the corresponding method obtained results with mean value most close to those from the LDCT images). It can be seen that the mean CT numbers of images from the three processed groups (2D SR, BM4D, 3D SR) are almost the same as those from the LDCT group. In aorta, the noise measurements of the 3D SR results are found lower than those of LDCT image, 2D SR results, BM4D results and even the SDCT images. In LCA, the noise measurements in 3D SR results are still lower than those in SDCT images and LDCT images, but slightly higher than those in 2D SR and BM4D results. In RCA, the noise measurement in 3D SR results are higher than all the other groups’ results except for LDCT image group. Since the mean values of all the groups are not significantly different (except for SDCT group), the SNR and CNR are basically inversely proportional to image noise.

The results show that compared to the other two methods, 3D SR method produced images with higher SNR and CNR in aorta, but lower SNR and CNR in LCA and RCA. However, this does not mean that 2D SR and BM4D methods can better handle anatomical details (LCA and RCA). Although the 2D SR and BM4D methods produced images with lower noise in LCA and RCA, the vessel details were generally over-smoothed and blurred (see Figs 4 and 5) in these regions. Besides, the 2D SR and BM4D methods were not effective in removing noise and artifacts in large flat regions. As a comparison, the proposed 3D SR method effectively removed the noise and artifacts in flat regions while giving a good preservation of the anatomical details.

### Qualitative Assessment

The qualitative assessment included 40 original images (20 LDCT images and 20 SDCT images), and 60 processed images (20 2D SR processed LDCT images, 20 BM4D processed LDCT images and 20 3D SR processed LDCT images). Half of the images were scanned under high-pitch mode and the other half under SAS mode. The quality of all the images were assessed using 4 subjective features: noise suppression, artifact suppression, contrast preservation and overall image quality using a 5-point subjective criterion (1 = unacceptable, 2 = substandard, 3 = acceptable, 4 = above average, 5 = excellent). Here, we define artifacts as any pattern influencing the diagnosis passively. Six radiologists (with averaged experience of 3.5 years) independently evaluated the randomized LDCT images, SDCT images, the 2D SR processed LDCT images, the BM4D processed LDCT images and the 3D SR processed LDCT images on a digital DICOM archiving/assessing workstation (ViewDEX 2.0^55^). The 4 subjective features were assessed for all the 100 images (40 original CT images, and 60 processed CT images), and this results in a total of 2400 ratings (100 × 4 × 6 = 2400). For each subset of images, the 4 categories of image scores were reported as means ± SDs (averaged scores of the 6 radiologists ± standard deviations) in Table 3.

The subjective quality scores of the original LDCT images and the processed LDCT images were compared with those of the corresponding SDCT images. Student’s t-test is used to evaluate if two sets of scores (scores of SDCT images and scores of LDCT/processed LDCT images) are significantly different from each other. For each set, the number of observation is 120 (20 images in each set assessed by 6 radiologists). Excel software (Microsoft) is used to run the Student’s t-test with P < 0.05 considered a statistically significant difference. Since the standard deviation of all the score sets are relatively similar, we used the t-test of two-samples assuming equal variances. The superscript * in Table 3 indicates that the labeled mean scores are significantly (P < 0.05) different from the mean scores for the original SDCT images.

As illustrated in Table 3, in general, the original LDCT images are found in lower subjective quality (except for contrast preservation) than the original SDCT images and the processed LDCT images. The 3D SR processed LDCT images obtained quality scores substantially higher than the 2D SR and BM4D processed LDCT images. Statistically significant differences (P < 0.05) with respect to the reference SDCT images can be noticed in all the subjective scores for the original LDCT images, 2D SR processed LDCT images and BM4D processed LDCT images. The subjective score differences between the 3D SR processed LDCT images and the original SDCT images are found not statistically significant (P>0.05) with regard to noise suppression and artifact suppression, but statistically significant in terms of contrast preservation and overall image quality. The reason that the contrast of 3D SR processed LDCT images are generally lower than SDCT images is that many low-contrast boundaries in LDCT images are already indistinguishable because of extensive noise. So as a post-processing method, it is difficult for the 3D SR method to restore these low-contrast boundaries based on already lost information. For this reason, the 3D SR method received lower subjective score than the SDCT’s score regarding overall image quality. Nonetheless, the 3D SR processed image group obtains scores most close to the results of SDCT group, compared with other groups.

### Computation Cost

Both the 2D SR method and the proposed 3D SR method include a dictionary training step and an OMP step. In our experiment, it takes 6.27 seconds to train a dictionary for the general 2D SR method. Since the dictionary size (512 × 1000) and the training set are much larger than 2D SR method, the training step in 3D SR processing is rather computational intensive and takes about 217.67 seconds. Fortunately, the dictionaries, once trained, can be used to process all the LDCT cases as demonstrated in the above experiments (refer also to^41^). Table 4 lists the average computation cost required in the operations following the dictionary training only. We can see that, with the trained dictionary available, the 2D SR method requires about 0.90 seconds (in average) to process one 512 × 512 slice; the BM4D method takes 3703.74 seconds (in average) to process one 512 × 512 × 270 CT data set (about 13.72 seconds per 2-D slice) and the 3D SR method requires 843.67 seconds (in average) to process one 512 × 512 × 270 CT data set (about 3.12 seconds per 2-D slice).

## Discussion and Conclusion

In this paper, we extended the 2D sparse representation based processing to the 3D domain to improve cardiac LDCT images. This 3D approach stems from the assumption that 3D CT slices convey coherent information on anatomical structures but noise and artifacts do not. Experimental results show that the 3D SR method can effectively suppress noise and artifacts without causing obvious image detail obscuring. The processed LDCT images can achieve an overall perceptual quality close to the corresponding SDCT images, with neither important structures lost nor obvious artifacts introduced. Low dose CT scans can be delivered to patient with the proposed post-processing method under LDCT scanning protocol. The radiation dose for a single patient can be reduced to less than 0.5 mSv under high-pitch mode and about 2.5 mSv under SAS mode while maintaining the image quality.

With no access to well-formatted raw data, the proposed processing can be easily applied to all the existing CT systems. Experiment results show that a pre-calculated global dictionary works well in implementing the proposed approach. We also found that the same parameter setting can be used in processing the LDCT images acquired from the same scan protocols, thus offering a tractable strategy for parameter determination.

Nonetheless, we also observe that some artifacts with strong intensity were hard to be suppressed without blurring some delicate tissue structures such as small vessels. Also, the whole computation cost of the 3D SR processing still needs a further acceleration to fulfill practical clinical requirements. Currently, some parameters still need to be empirically set. To obtain a better performance of artifact suppression, combinations with statistically iterative algorithms or some multiscale artifact-suppressing constraints should be considered^31^,^56^,^57^,^58^,^59^. A full evaluation of the proposed processing should also include testing the segmentation accuracy enhancement that might be potentially brought by the proposed method^60^. Future work will be devoted to address these issues.

## Additional Information

**How to cite this article**: Shi, L. *et al*. Improving Low-dose Cardiac CT Images based on 3D Sparse Representation. *Sci. Rep*. **6**, 22804; doi: 10.1038/srep22804 (2016).

## Figures and Tables

**Table 1:** Parameter setting for 2D and 3D sparse representation based methods.

Parameters	2D SR processing	3D SR processing
Block size 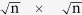	8 × 8	8 × 8 × 8
Dictionary size *n* × *K*	64 × 256	512 × 1000
Atom sparsity (Sparse K-SVD) *L*	6	16
Lagrange multiplier 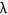	30	30
Tolerance parameter 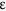	7.62 × 10^4^	4.98 × 10^5^
Step size	1	2

**Table 2:** Image Quantitative Scores (mean ± SDs, BOLD fonts represent the best performance among the three methods).

Quantitative Metrics	SDCT images	LDCT images	2D SR results	BM4D results	3D SR results
Mean CT number (HU)					
Aorta	436.25 ± 61.78	448.18 ± 85.37	**448.60** ± 85.17	448.71 ± 85.41	449.08 ± 85.29
LCA	435.32 ± 52.43	435.01 ± 69.79	431.14 ± 72.96	429.72 ± 71.95	**432.96** ± 72.04
RCA	444.04 ± 56.91	465.01 ± 85.56	463.27 ± 85.71	462.34 ± 84.26	**465.42** ± 84.10
Perivascular fat	−80.11 ± 17.99	−75.79 ± 14.93	−74.60 ± 15.58	**−74.89** ± 16.79	−73.35 ± 13.77
Image noise (HU)					
Aorta	18.69 ± 3.33	32.23 ± 8.88	20.12 ± 6.78	18.98 ± 6.24	**15.18** ± 5.34
LCA	19.77 ± 11.60	18.95 ± 5.27	10.19 ± 2.70	**9.93** ± 4.26	12.22 ± 5.57
RCA	12.72 ± 5.34	20.75 ± 6.43	13.70 ± 5.74	**11.88** ± 4.95	16.55 ± 6.20
Perivascular fat	18.32 ± 9.80	29.13 ± 2.70	17.43 ± 3.07	15.60 ± 3.41	**14.14** ± 2.91
SNR					
Aorta	23.64 ± 3.68	14.76 ± 4.99	24.29 ± 8.99	25.93 ± 10.98	**33.03** ± 15.29
LCA	27.73 ± 14.56	24.21 ± 7.05	43.61 ± 9.95	**50.38** ± 24.67	42.97 ± 24.03
RCA	38.30 ± 10.82	23.75 ± 6.52	39.20 ± 17.90	**42.66** ± 12.47	30.22 ± 7.96
CNR					
Aorta	27.95 ± 4.25	17.18 ± 5.40	28.16 ± 9.68	30.03 ± 11.77	**38.14** ± 16.52
LCA	32.59 ± 16.54	28.40 ± 8.12	51.04 ± 10.68	**59.57** ± 30.69	50.57 ± 29.32
RCA	45.15 ± 12.56	27.55 ± 7.13	45.37 ± 20.27	**49.58** ± 14.26	35.01 ± 9.11

**Table 3:** Image Qualitative Scores (mean ± SDs, BOLD fonts represent the best performance among the three methods).

	SDCT	LDCT	2D SRprocessedLDCT	BM4DprocessedLDCT	3D SRprocessedLDCT
Noise Suppression	4.08 ± 0.85	2.86 ± 0.75*	3.55 ± 1.08*	3.63 ± 0.86*	4.02 ± 0.73
Artifact Suppression	4.10 ± 0.67	3.10 ± 0.73*	3.36 ± 0.93*	3.24 ± 0.87*	3.96 ± 0.71
Contrast Preservation	4.22 ± 0.58	3.75 ± 0.65*	3.44 ± 0.83*	3.38 ± 0.72*	3.89 ± 0.75*
Overall Image Quality	4.23 ± 0.64	3.18 ± 0.67*	3.36 ± 0.92*	3.27 ± 0.81*	4.08 ± 0.76*

*Significantly (*P* < 0.05) means different from the mean scores for the original SDCT images.

**Table 4:** The average computation cost (in seconds) for the 2D SR method in ref. 45, the bm4d method in ref. 54 and the proposed 3D SR method.

	2D SR	BM4D	3D SR
For a whole dataset	243.35	3703.74	843.67
For one slice	0.90	13.72	3.12
