# Corrigendum: Improving Low-dose Cardiac CT Images based on 3D Sparse Representation

**DOI:** 10.1038/srep24594

**Published:** 2016-04-22

**Authors:** Luyao Shi, Yining Hu, Yang Chen, Xindao Yin, Huazhong Shu, Limin Luo, Jean-Louis Coatrieux

Scientific Reports 6: Article number: 2280410.1038/srep22804; published online: 03162016; updated: 04222016

In the original version of this Article, there were typographical errors in Affiliation 3 which was incorrectly listed as ‘Department of Radiology, Nanjing Hospital Affiliated to Nanjing Medical University, 210096, Nanjing, China.’ The correct affiliation is listed below:

Department of Radiology, Nanjing First Hospital, Nanjing Medical University, 210006, Nanjing, China.

These errors have now been corrected in the PDF and HTML versions of the Article.

